# Physical fitness status modulates the inflammatory proteins in peripheral blood and circulating monocytes: role of PPAR-gamma

**DOI:** 10.1038/s41598-020-70731-6

**Published:** 2020-08-24

**Authors:** Barbara Moura Antunes, José Cesar Rosa-Neto, Helena Angélica Pereira Batatinha, Emerson Franchini, Ana Maria Teixeira, Fábio Santos Lira

**Affiliations:** 1 Exercise and Immunometabolism Research Group, Postgraduation Program in Movement Sciences, Department of Physical Education, Universidade Estadual Paulista (UNESP), Presidente Prudente, SP 19060-900 Brazil; 2 Immunometabolism Research Group, Institute of Biomedical Sciences, University of São Paulo (USP), São Paulo, SP Brazil; 3 School of Physical Education and Sport, University of São Paulo (USP), São Paulo, Brazil; 4 Research center for sport and physical activity, Faculty of sport sciences and physical education, University of Coimbra, Coimbra, Portugal

**Keywords:** Metabolism, Cytokines, Inflammation

## Abstract

The aim of this study was to analyze the metabolic and molecular profile according to physical fitness status (Low or High VO_2max_) and its impacts on peripheral and cellular inflammatory responses in healthy men. First (*Phase I)*, inflammatory profile (TNF-α, IL-6, IL-10) was analyzed at baseline and post-acute exercise sessions performed at low (< 60% VO_2max_) and high (> 90% VO_2max_) intensities considering the individual endotoxin concentrations. Next (*Phase II),* monocyte cell cultures were treated with LPS alone or associated with Rosiglitazone (PPAR-γ agonist drug) to analyze cytokine production and gene expression. Monocyte subsets were also evaluated by flow cytometry. A positive relationship was observed between LPS concentrations and oxygen uptake (VO_2max_) (r = 0.368; p = 0.007); however, in the post-exercise an inverse correlation was found between LPS variation (Δ%) and VO_2max_ (r = -0.385; p = 0.004). With the low-intensity exercise session, there was inverse correlation between LPS and IL-6 concentrations post-exercise (r = -0.505; p = 0.046) and a positive correlation with IL-10 in the recovery (1 h post) (r = 0.567; p = 0.011), whereas with the high-intensity exercise an inverse correlation was observed with IL-6 at pre-exercise (r = -0.621; p = 0.013) and recovery (r = -0.574; p = 0.016). When monocyte cells were treated with LPS, High VO_2max_ individuals showed higher PPAR-γ gene expression whereas Low VO_2max_ individuals displayed higher IL-10 production. Additionally, higher TLR-4, IKK1, and PGC-1α gene expression were observed in the High VO_2max_ group than Low VO_2max_ individuals. In conclusion, even with elevated endotoxemia, individuals with High VO_2max_ exhibited higher IL-6 concentration in peripheral blood post-acute aerobic exercise and lower IL-10 concentration during recovery (1 h post-exercise). The anti-inflammatory effects linked with exercise training and physical fitness status may be explained by a greater gene expression of IKK1, TLR-4, and PGC-1α, displaying an extremely efficient cellular framework for the PPAR-γ responses.

## Introduction

Higher endotoxin (or lipopolysaccharide—LPS) concentration in the blood is positively associated with a sedentary lifestyle^1^ and Western diet^2,3^, acting as a major trigger for increasing the production of inflammatory cytokines by immune cell activation. The signaling pathway that orchestrates the immune response in mononuclear cells, especially monocytes and macrophages, occurs through activation of *toll like receptors (TLR)* and transcriptional factors, mainly TLR-4 and nuclear factor *kappa* B (NF-kB)^4,5^. This environment is favorable for the installation and development of metabolic disorders as obesity, diabetes *mellitus* type 2, cardiovascular diseases, and others^6,7^.

Some training protocols, as short-term strenuous exercise (i.e., progressive test with volitional exhaustion)^8^, long duration (i.e., ultra-distance triathlon)^9^, or performed at higher intensities under heat stress^10^, are able to increase the plasmatic LPS concentrations due to increased permeability of the gastrointestinal barrier^11,12^. However, well-trained individuals exhibit smaller increases in LPS concentration front of exercise, when compared with untrained individuals, suggesting a positive modulation mediated by physical fitness status^9,13^.

There is an inflammatory scenario inherent to physical exercise suggesting that the process of injury and tissue repair is composed of three inflammatory waves. In the first two inflammatory phases, there are greater recruitment and activity of neutrophils, lymphocytes CD8^+^ and M1 macrophages (pro-inflammatory) and, through the NF-kB pathway, increased inflammatory cytokine synthesis (i.e., TNF-α, IL-6, IL-1β, IFN-γ). Subsequently, the anti-inflammatory phase is marked by the increase of lymphocytes Treg and the microenvironment conversion from pro to anti-inflammatory, given the phenotypic change of macrophages M1 to M2 (anti-inflammatory) and, consequently, increased synthesis of IL-10, IL-1ra, IL-4, IL-13, via PPAR-γ^14^.

In this sense, exercise training is strongly suggested as a potential anti-inflammatory strategy given that it is able to counter-regulate inflammation by increasing anti-inflammatory biomarkers^15^. Muscle contraction, mediated by exercise training, produces and releases myokines, especially IL-6^16,17^, that has pleiotropic function and acts as a trigger factor for anti-inflammatory cytokines release, showing a central role in the cascade activation of IL-10 and IL-1ra^18,19^.

Besides anti-inflammatory responses, IL-6 is also associated with energetic metabolism during exercise training sessions given that this myokine is capable to act as energetic sensor in response to decreased glycogen stores in the skeletal muscle acting mainly in the liver and adipose tissue to increase the bioavailability of energy substrate by glucose metabolism regulation and lipolysis stimulation^20,21^.

A previous study conducted by our group showed that exercise performed at higher intensities (> 60% VO_2max_) exhibited anti-inflammatory responses, mainly through IL-10 production and this response was physical fitness-dependent^22^. In this perspective, two molecular pathways are related with anti-inflammatory response together with the metabolic reprogramming in myeloid cells. On the one hand AMP-activated protein kinase (AMPK), a kinase energetic sensor, induces the oxidative pathway in immune cells, promoting an anti-inflammatory profile in macrophages and lymphocytes, chronically^23^.

On the other hand, the peroxisome proliferator-activated receptor gamma (PPAR-γ), with its co-activators as peroxisome proliferator-activated receptor gamma coactivator-1 alpha (PGC-1α), are anti-inflammatory factors and seem to orchestrate the positive responses linked with exercise training and physical fitness status^24,25^. Silveira and colleagues^26^ found a pro-inflammatory profile in sedentary animals with PPAR-γ deletion in macrophages while exercise training leads for diminished inflammation in adipose tissue.

LPS and PPAR-γ have capacity to modulate the inflammatory response, although in antagonistic ways, given that PPAR-γ counteracts the LPS response. We hypothesized that physical fitness status would be a crucial factor to obtain the anti-inflammatory responses. Thus, the purposes of the present study were (1) to analyze the inflammatory and metabolic responses, according to individual physical fitness status, after acute aerobic exercise sessions and (2) to investigate molecular mechanisms that regulate peripheral inflammatory responses.

## Material and methods

### Participant recruitment

The present study comprised two phases (*Phase I and Phase II*). In *phase I,* 28 healthy male individuals (age: 28.8 ± 5.6 years; body mass: 75.8 ± 9.9 kg; VO_2mean_: 50.5 ± 8.8 mL kg^−1^ min^−1^) were recruited to participate in the study in order to analyze the peripheral inflammatory profile at baseline and in response to two acute-exercise sessions according to endotoxemia. In *Phase II*, another 22 healthy male individuals (age: 25.8 ± 5.7 years, body mass: 76.5 ± 14.4 kg, VO_2mean_: 47.8 ± 12.3 mL kg^−1^ min^−1^) were recruited in order to conduct molecular analysis of monocyte cell cultures treated for 24 h.

Initially, all participants were classified as physically inactive, physically active or well-trained using the International Physical Activity Questionnaire (IPAQ). All participants were required to complete all exercise sessions. This study was approved by the local research ethics committee of the Sao Paulo State University "Júlio de Mesquita Filho" and duly registered in Brazil Platform (national electronic system created by the Federal Government to systematize the receipt of research projects involving human beings in Ethics Committees throughout the country) (CAAE: 31168714.6.0000.5402) and the research was conducted according to the 2013 Revision of the Declaration of Helsinki. Healthy men were included, without any health disorders, such as inflammatory, cardiorespiratory and osteoarticular diseases, and who had not used any ergogenic substances or medicines for at least six months prior to the study. Written informed consent was obtained from all participants prior to participation.

### Maximal incremental test and aerobic exercise bouts

For *Phases* I and II, all participants completed a maximal incremental test on a cycle ergometer (Inbrasport CG-04, Embramed, Porto Alegre, Brazil) to determine maximal oxygen uptake. The initial workload was 35 watts for sedentary individuals, 70 watts for the physically active, and 105 watts for well-trained individuals, with an increase of 25 watts every 3-min, and under constant speed (70–90 rpm) until exhaustion^27^. Voluntary exhaustion criteria were gas exchange ratio > 1.1, HR_max_ > 90% of the maximum expected for age, and rating of perceived exertion (RPE) > 18. The maximum workload (W_max_) and maximal oxygen uptake (VO_2max_) were assessed by a breath-by-breath gas analyzer (Quark PFT, Cosmed, Rome, Italy). In *Phase* I, ventilatory thresholds (aerobic and anaerobic thresholds) were determined by the VE∙VO_2_^–1^ vs. workload and VE∙VCO_2_^–1^ vs. workload, as suggested by Binder and colleagues^28^, to prescribe acute-exercise sessions at low and high-intensities.

In *Phase I,* aerobic acute-exercise sessions started with 5 min warm-up on a cycle ergometer at 30% of W_max_ for all intensities. Randomly, two sessions (with at least 48 h interval between the sessions) were performed at low (< 60% VO_2max_—90% of aerobic threshold) and high (> 90% VO_2max_—midpoint between anaerobic threshold and W_max_) intensities until exhaustion or up to 60 min^28^.

### Blood collection and Isolation of human peripheral mononuclear cells

In both *Phases,* blood samples were collected by peripheral puncture of a forearm vein. In *Phase* I, at baseline (1.5 h after breakfast and immediately before exercise sessions), immediately post-session (post-exercise), and recovery (60 min after the end-session). Blood sample treatment were processed as previously described by the authors^22^. In *Phase* II, the peripheral mononuclear cell isolation, mediated by adherence protocol in order to obtain the monocytes, was carried out as previously conducted by the authors^29^. A standard breakfast was offered respecting the 25% of total energy value and the recommended macronutrients proportion as previous mentioned^29^.

### Flow cytometry

For phenotyping, the peripheral blood mononuclear cells (PBMCs) (1 × 10^6^) were labeled with antibodies and fluorochromes specific for CD14 (APC Mouse Anti-Human CD14; BD Biosciences) and CD16 (PE Mouse Anti-Human CD16; BD Biosciences). Samples were run on a FACScalibur flow cytometer (BD Biosciences, Mississauga, ON, Canada), using Cell Quest software, and analyzed using Infinicyt software, version 1.7 (Cytognos, Salamanca, Spain). Briefly, peripheral blood cells (PBCs) were labeled and incubated for 30 min at room temperature in the dark. After this, 500 ul of PBS was added and centrifuged (5 min at 1500 rpm) for washing, and the supernatant discarded. The cells were resuspended in 500 ul saline solution, and immediately acquired in a flow cytometer.

### Monocytes culture

A total of six individual wells with 1 mL of final volume were treated for 24 h at 37 °C and 5% CO2. Monocyte cells were cultured with TLR-4/NF-kB agonist (LPS) and PPAR-γ agonist drugs. In the treatment was used LPS ([100 ng/mL]) and Rosiglitazone ([1 μM]) (both of the Sigma-Aldrich Co. LLC-St. Louis, MO, USA) in isolate or conjugate (LPS + Rosiglitazone) forms with the same final drug concentration in order to stimulate the inflammatory (IKK/NF-kB) and anti-inflammatory (PGC-1α/PPAR-γ) signaling pathway, respectively. After 24 h, the supernatants were collected and stored at − 80 °C for cytokine measurement and the adhered cells were treated with Brazol (LGC Biotechnology Ltda.—Cotia/SP) for gene expression analysis by real-time PCR.

### Endotoxin concentration and cytokine measurements

In *Phase* I, endotoxin concentrations were analyzed pre and post-exercise sessions by a chromogenic limulus amebocyte lysate (LAL) test, which is a quantitative test for gram-negative bacteria endotoxin (LAL Kit; QCL-1000, Lonza, Lonza Walkersville Inc- Walkersville, MD 21793) using lyophilized endotoxin (*E. coli* origin) for the standard curve. The concentration of endotoxin is linear in the range of 0.1–1.0 EU/mL. TNF-α, IL-6, and IL-10 concentrations were determined in peripheral blood (*Phase* I) and monocyte culture supernatant (*Phase* II) by the enzyme-linked immunosorbent assay (ELISA) technique using high detection sensitivity kits (R&D System, a biotechne brand, Quantikine ELISA, Inc., Minneapolis, USA) with ranges between 15.6–1,000 pg/mL for TNF-α, 3.13–300 pg/mL for IL-6, and 7.8–500 pg/mL for IL-10; and intra- and inter-assay variations (%) of 4.2–5.2 and 4.6–7.4 for TNF-α, 1.6–4.2 and 3.3–6.4 for IL-6, and 1.7–5.0 and 5.9–7.5 for IL-10, respectively.

### RNA isolation and RT-PCR assays

Total monocyte RNA was extracted with Brazol reagent (LGC Biotechnology Ltda.—Cotia/SP) following the manufacturer's recommendations and for the RT-PCR analyzes 12 volunteers were used. Reverse transcription to complementary DNA (cDNA) was performed using the High Capacity cDNA Reverse Transcription kit (Applied Biosystems—Thermo Fisher Scientific, Foster, CA). The generated cDNA was stored at − 80 °C for further analysis (NF-kB, IKK1, TLR-4, HIF-1α, PPAR-γ, PGC-1α, and AMPK) by RT-PCR with Power SYBR Green PCR Master Mix (Applied Biosystems). Primer sequences are shown in the Table 1. Quantification of gene expression was carried out using the glyceraldehyde3‐phosphate dehydrogenase gene (GAPDH) as an internal control. Relative quantification of genes of interest was calculated using the 2^−ΔΔCT^ formula, in which CT is the difference between the cycle threshold (CT) value for the gene of interest and CT value for the housekeeping gene.

**Table 1:** Primer sequences of RT-PCR analysis.

Gene	Primer forward	Primer reverse
GAPDH	ACAACTTTGGTATCGTGGAAGG	GCCATCACGCCACAGTTTC
PPAR-γ	CAGCCTTTAACGAAATGACCA	TGTGGAGTAGAAATGCTGGA
NF-KB	GAAGCACGAATGACAGAGGC	GCTTGGCGGATTAGCTCTTTT
TLR-4	TTTATCCAGGTGTGAAATCCAG	AGATGCTAGATTTGTCTCCACAG
PGC-1ɑ	GCAGACCTAGATTCAAACTCAG	GTATTCGCCATCCCTCTGTC
IKK1	GACCTTCAGATCACTCCTACAG	CAAATGACCAAACAGCTCCT
CD36	TTTGGCTTAATGAGACTGGGAC	AAAGCAACAAACATCACCACAC
L-CAT	AAGCTGGACAAACCAGATGTG	TAGACAACCCTGGTGTTATCG
CETP	AAATCTTCCAAGAGGTTGTCGG	CCATCACTGAAGAATTGACCAC
HIF-1α	AGTTCACCTGAGCCTAATAGTCC	TCCAAGTCTAAATCTGTGTCCTG
AMPK	GGCACGCATACCCTTGAT	TCTTCCTTCGTACACGCAAATAA

### Statistical analysis

Data normality was verified using the Shapiro–Wilk test and a non-parametric analysis was adopted for peripheral variables of *Phase I*, as one or more parameters followed a non-parametric distribution, and for the *Phase II* was adopted parametric analyses given the normal distribution of data. In *Phase* I, to compare the LPS concentration according to physical fitness, all subjects were classified according to LPS mean values (low or high endotoxemia) and the comparison of the physical fitness was conducted by the Mann–Whitney test. Additionally, the relationship between physical fitness and LPS concentration (at baseline and post-exercise sessions) as well as the relationship between LPS release and cytokine production at low and high intensity exercise sessions was verified through Spearman correlation. In *Phase* II, to compare the groups according to physical fitness (low and high VO_2max_), the Student's t-test for independent samples was used. Differences in cytokine concentration, in the monocyte culture supernatant, as well as the gene expression, between the physical fitness groups exposed to different stimulus (LPS, Rosiglitazone, and LPS + Rosiglitazone) were analyzed by ANOVA two-way (group X treatment), and when an interaction (treatment x group) was observed, a Bonferroni post hoc test was conducted and the partial eta-square for ANOVA was presented for main effect of time (*η*^*2*^). Statistical significance was set at P < 0.05 and the data were analyzed using the Statistical Package for Social Sciences 22.0 (*SPSS* Inc. Chicago. IL. USA).

## Results

### Relationship between endotoxemia, physical fitness status and exercise sessions

**Figure 1:**
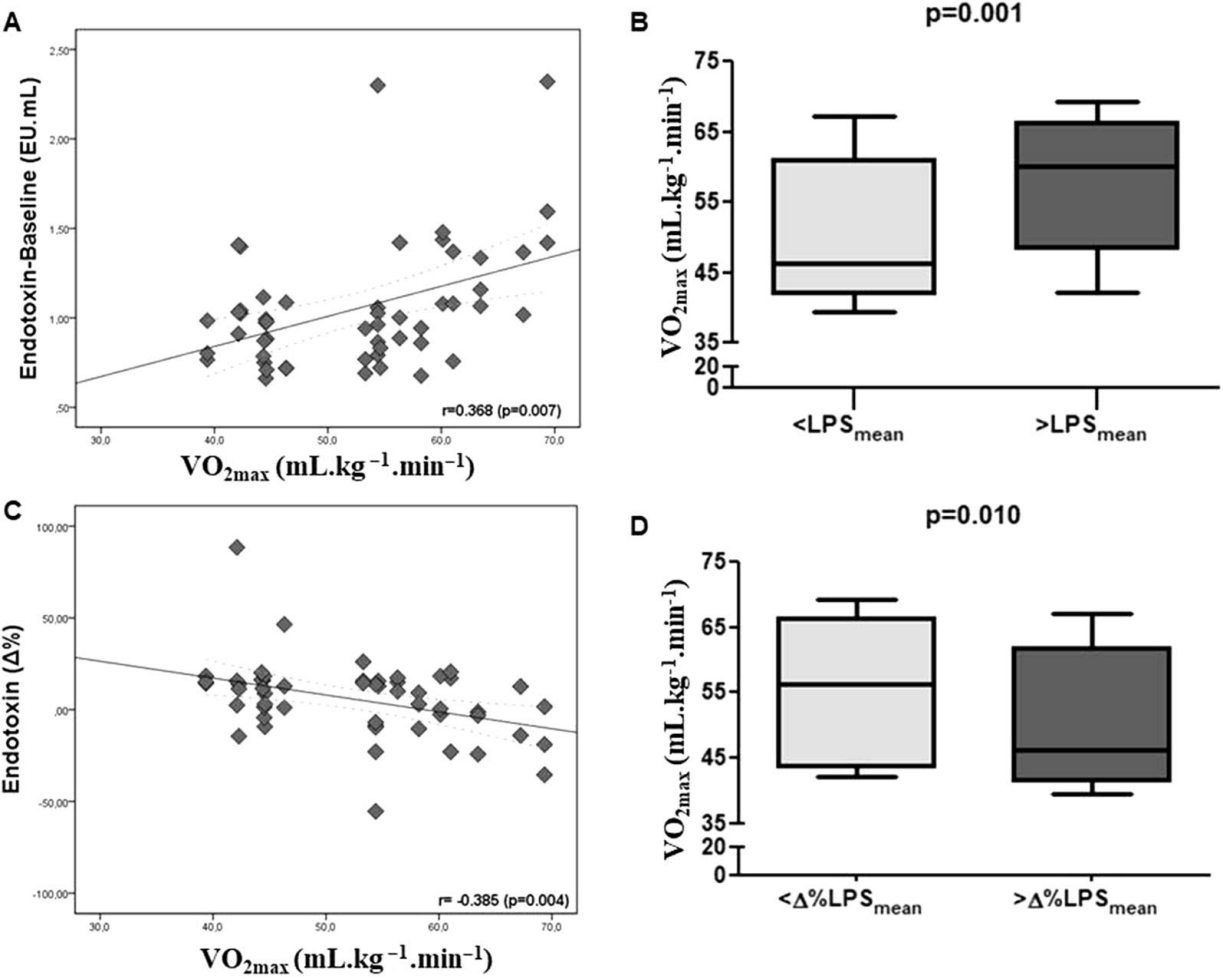
Relationship between LPS concentration (absolute and relative variation) and physical fitness level, represented by VO_2max_, at baseline (**A**, **B**) and immediately post exercise-session (**C**, **D**), independently of exercise intensities (all N = 28 subjects; < LPS N = 14; > LPS N = 14). **(A),** **(C)** represent Spearman's correlation with significance established at 5%; **(B)**, **(D)** represent Mann–Whitney test expressing the results in median and interquartile range being the Y-axis represented by maximal oxygen uptake and X-axis represented by LPS initial concentration or variation post-exercise.

**Figure 2:**
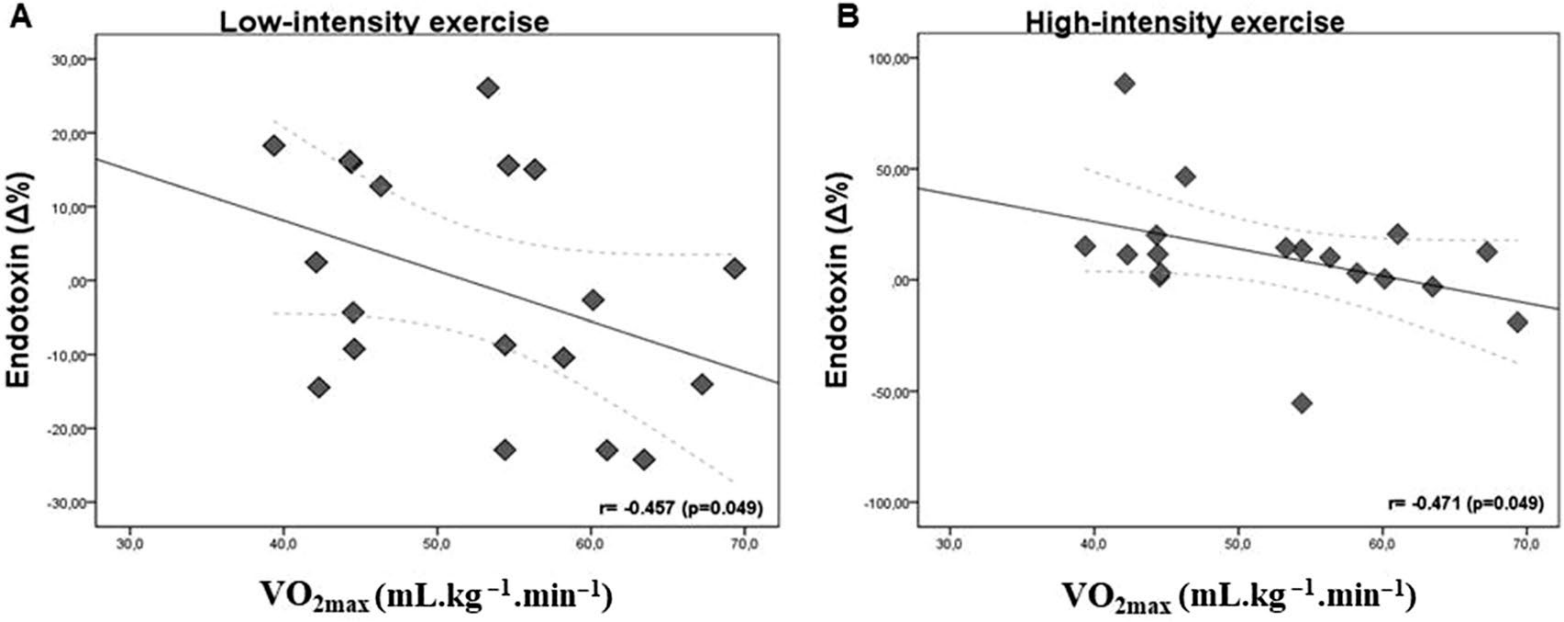
Relationship between relative variation in LPS (percentage difference between the pre and immediately post-exercise concentration values) and physical fitness status, represented by VO_2max_, according to exercise sessions performed at low and high intensities (all N = 28 subjects; < LPS N = 14; > LPS N = 14). **(A)**, **(B)** represent Spearman's correlation with significance established at 5%.

In general, we identified a positive relationship between post-prandial LPS concentration and VO_2max_, independently of intensities of exercise sessions, as well as association between LPS and cytokine concentrations, mainly IL-6 and IL-10 post-exercise. The Fig. 1A–D shows the relationship between LPS concentration and physical fitness status represented by VO_2max_. At baseline, a positive correlation was observed between LPS concentration and VO_2max_ (Fig. 1A) (p = 0.007) and, when grouping the subjects according to LPS concentrations [< or > LPS_mean_ (Baseline_mean_: 1.05 EU/mL)], individuals with greater VO_2max_ (VO_2mean_: 58.3 ± 8.9 mL kg^−1^ min^−1^) presented higher LPS concentrations (LPS_mean_: 1.39 ± 0.36 EU/mL) when compared with individuals with lesser VO_2max_ (VO_2mean_: 49.5 ± 7.2 mL kg^−1^ min^−1^/LPS_mean_: 0.86 ± 0.12) (p = 0.001) (Fig. 1B). Importantly, post-exercise and independently of exercise intensities, an inverse correlation was observed between LPS variation (Δ%) and physical fitness level (Fig. 1C) (p = 0.034) and, when the subjects were grouped according to Δ%LPS (< or > Δ%LPS_mean_ (Post-exercise_mean_: 5.55%)), individuals with greater VO_2max_ (VO_2mean_: 56.0 ± 9.1 mL kg^−1^ min^−1^) presented lower LPS variation (Δ%LPS: − 10.5%/LPS_mean_: 1.05 ± 0.29 EU/mL) when compared with individuals with lesser VO_2max_ (VO_2mean_: 49.9 ± 7.8 mL kg^−1^ min^−1^/Δ%LPS: 18.8%/LPS_mean_: 1.10 ± 0.29) (p = 0.010) (Fig. 1D). The same inverse relationship between LPS variation (Δ%) and physical fitness status, according to exercise intensities, was observed at lower intensity, in the group with greater VO_2max_ after exercise sessions performed at low (p = 0.049) and high (p = 0.049) intensities (Fig. 2).

### Correlation between endotoxemia mediated by exercise sessions and peripheral cytokine concentrations

The relationship between LPS concentration post-exercise and cytokine productions after low and high intensity exercises is shown in Table 2. At low-intensity exercise there was an inverse correlation between LPS release and IL-6 immediately post-exercise (p = 0.046) and a positive correlation with IL-10 concentration in the recovery (p = 0.011), whereas, at high-intensity exercise, an inverse correlation was observed with IL-6 concentration at pre-exercise (p = 0.013) and recovery period (p = 0.016).

**Table 2:** Relationship post-exercise between LPS and cytokines production after low and high intensity exercises.

	Low-intensity exercise			High-intensity exercise		
	Pre-exercise	Post-exercise	Recovery (60 min)	Pre-exercise	Post-exercise	Recovery (60 min)
TNF-α (pg mL^−1^)	r = − 0.333 (0.164)	r = − 0.091 (0.710)	r = 0.082 (0.738)	r = − 0.184 (0.480)	r = − 0.165 (0.512)	r = − 0.207 (0.409)
IL-6 (pg mL^−1^)	r = − 0.409 (0.092)	r = − 0.505 (0.046)*	r = − 0.094 (0.729)	r = − 0.621 (0.013)*	r = − 0.335 (0.204)	r = − 0.574 (0.016)*
IL-10 (pg mL^−1^)	r = 0.088 (0.721)	r = 0.144 (0.556)	r = 0.567 (0.011)*	r = − 0.095 (0.708)	r = 0.163 (0.518)	r = 0.133 (0.598)

r = rho value of Spearman correlation.

### Influence of LPS stimulation on activation or inhibition of PPAR-γ according to physical fitness status

**Figure 3:**
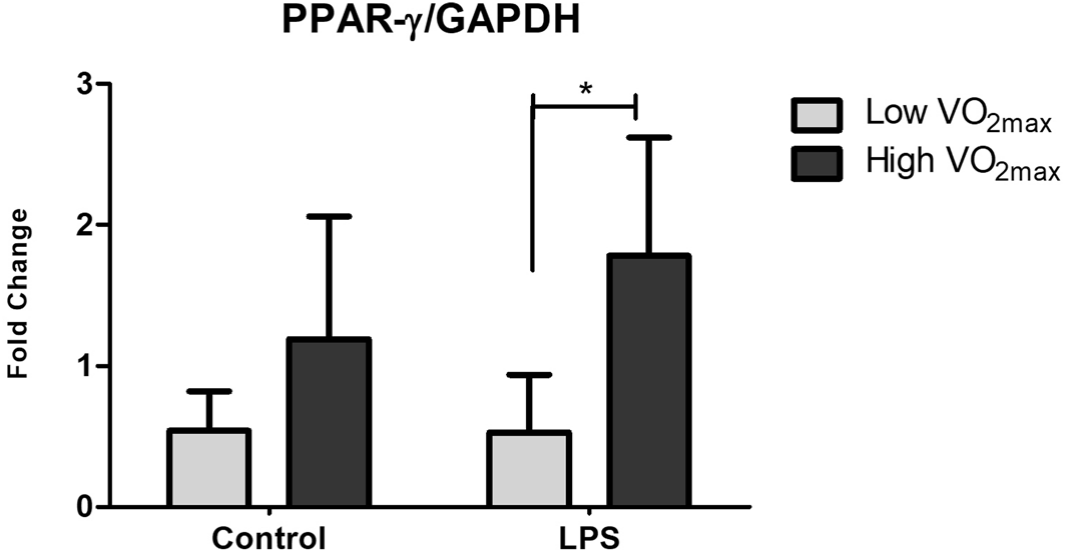
Relative Peroxisome proliferator-activated receptor gamma (PPAR-γ) gene expression in monocytes cell culture treated with LPS ([100 ng/mL]) and without stimulation (control) of individuals classified as Low (N = 6) or High (N = 6) VO_2max._ (*)Significant difference between groups (p < 0.05).

In order to investigate the influence of LPS on activation or inhibition of PPAR-γ the monocyte culture was performed in the presence and absence of LPS for 24 h. Figure 3 shows the gene expression of PPAR-γ with or without LPS stimulation according to physical fitness status (Low or High VO_2max_) with no statistical differences found in the control treatment when compared the fitness groups; however, when compared the fitness groups in response to LPS treatment, the High VO_2max_ group demonstrated a significant increase (p = 0.009) in PPAR-γ mRNA.

### Monocyte genotype expression by RT-PCR analyzes

**Figure 4:**
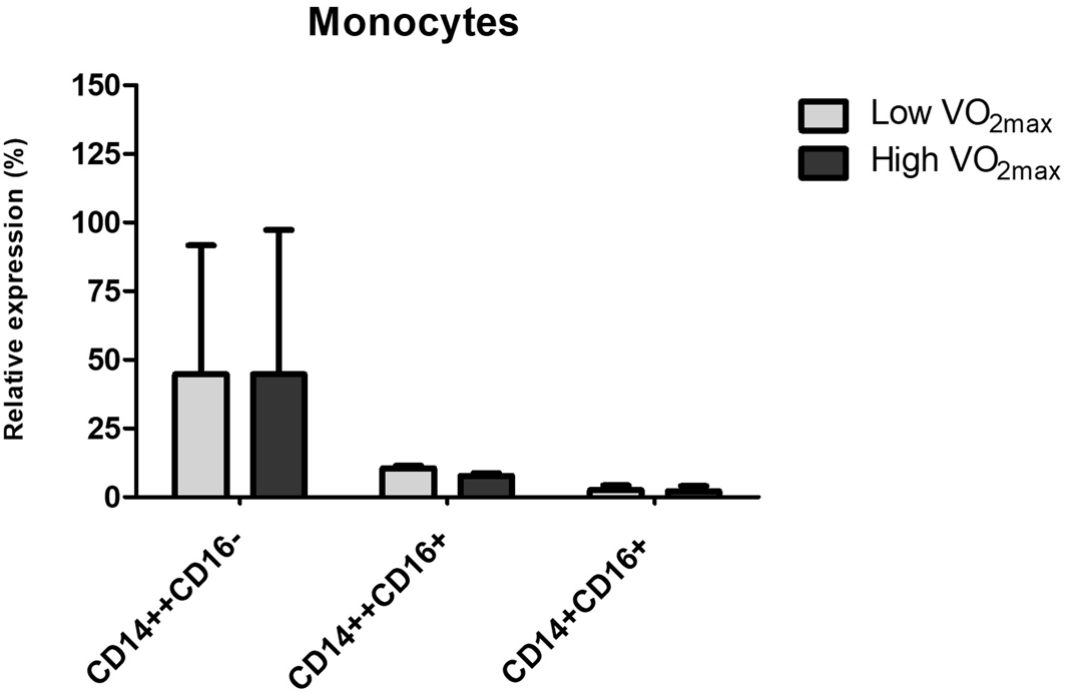
Monocyte population in peripheral blood mononuclear cells (PBMC) of individuals classified as Low (N = 11) or High (N = 11) VO_2max._ The data are showing in relative expression (percentage of expression) according to the subpopulation.

**Figure 5:**
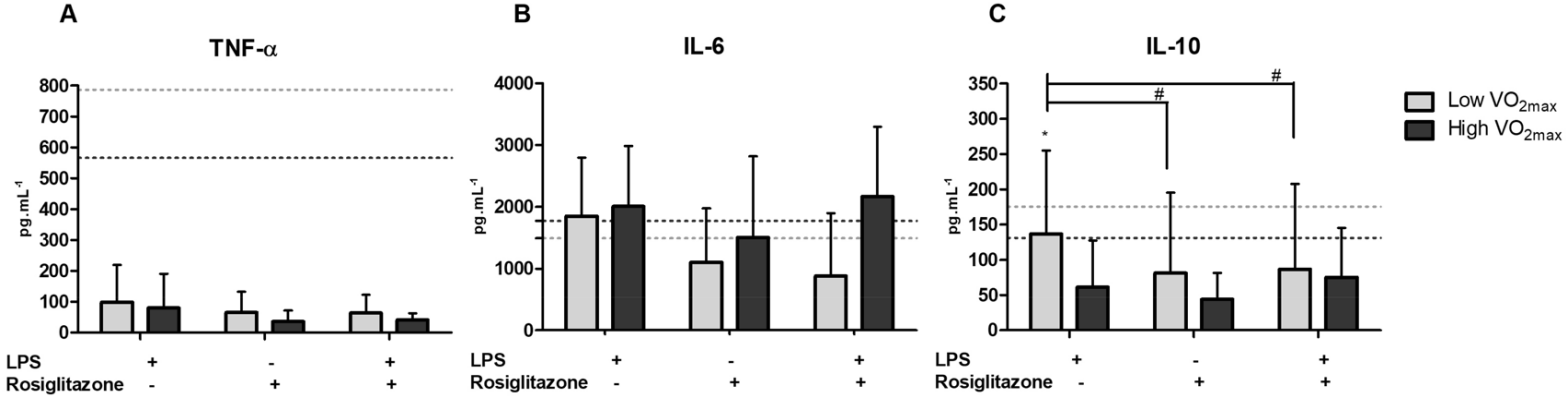
Cytokine release in monocyte cell culture incubated for 24 h with ( +) or without (−) LPS and Rosiglitazone (Low VO_2max_ N = 11 or High VO_2max_ N = 11). Values are expressed as mean ± standard deviation. *Group X Treatment interaction, #Difference within the group between the treatments. Dashed line represents the control group (without stimuli) according to groups. Significance value adopted was 5%.

**Figure 6:**
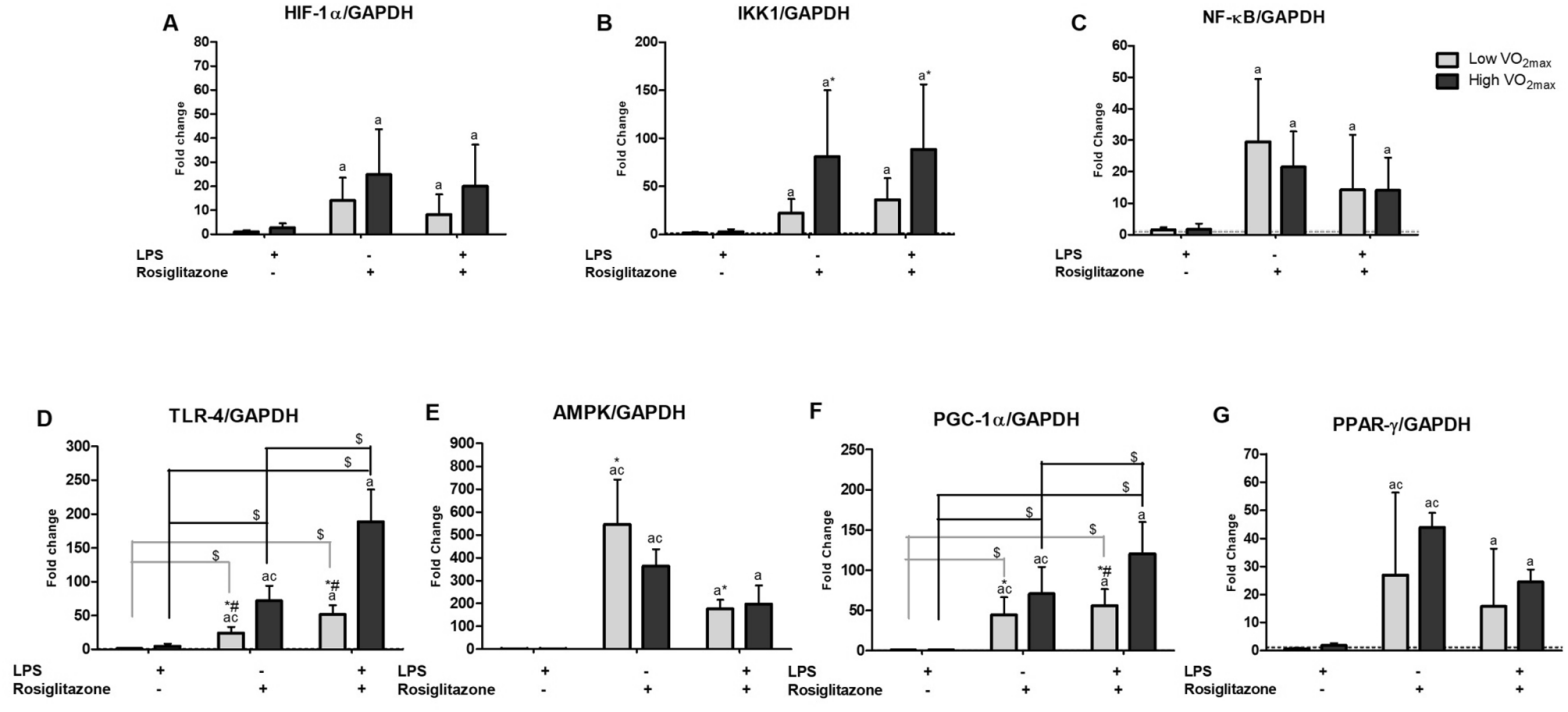
Relative gene expression of monocyte cell culture incubated for 24 h with ( +) or without (-) LPS and Rosiglitazone (Low VO_2max_ N = 6 or High VO_2max_ N = 6). Values expressed as mean ± standard deviation. Letter = difference of condition (treatment) independent of the group (physical fitness status) and Symbols = difference of condition (treatment) dependent of the group (physical fitness status).^a^Different from control, ^b^Different from LPS, ^c^Different from LPS + Rosiglitazone, *Group effect, #Group X Treatment interaction, $Difference within the group between the treatments. Dashed line represents the control group (without stimuli) according to groups. Significance value adopted was 5%.

Figure 4 shows the phenotypic monocytes distribution according to physical fitness status (Low or High VO_2max_). No differences were identified in the CD14^++^CD16^-^ (p = 0.418), CD14^++^CD16^+^ (p = 0.541), or CD14^+^CD16^+^ (p = 0.674) subsets; however, when analyzing the cytokine production on monocyte culture supernatant treated with PPAR-γ agonist (Fig. 5) and gene expression of proteins related to PPAR-γ and NF-kB activity modulation (Fig. 6), significant differences were detected.

### Molecular analyzes from monocyte cell culture

With respect to cytokine production in monocyte culture supernatant, no effects of treatment and physical fitness status for TNF-α concentration were observed. For IL-6, there was a tendency (*F* = 3.107, *p* = 0.063, *ƞ*^*2*^: 0.206) to higher production in LPS when compared with Rosiglitazone treated monocyte cell cultures. For IL-10, there was a main effect of treatment (*F* = 5.071, *p* = 0.011, *ƞ*^*2*^: 0.211) with higher concentration under LPS stimulation, when compared with Rosiglitazone, and statistically significant interactions (*F* = 4.028, *p* = 0.026, *ƞ*^*2*^*: *0.175); however, the post-hoc showed a trend of difference (*p* = 0.08) between groups under LPS treatment. It is important to highlight that the Low VO_2max_ group had higher IL-10 concentrations under LPS stimulation when compared with the other treatments.

When analyzing the gene expression, hypoxia-inducible factor-1 alpha (HIF-1α) showed a main effect of treatment (*F* = 15.904, *p* < 0.001, *η*^*2*^: 0.614) with lower expression under LPS stimulation when compared with both other treatments and a trend for a difference between groups (*p* = 0.07) under Rosiglitazone and LPS + Rosiglitazone treatments. For IkappaB kinase 1 (IKK1), there was a main effect of treatment (*F* = 7.656, *p* = 0.003, *η*^*2*^: 0.434) with lower expression under LPS stimulation, compared with both other treatments, and significant differences between groups (*F* = 6.911, *p* = 0.025, *η*^*2*^*: *0.409), with lower IKK1 expression in the Low VO_2max_ group. For NF-kB there was a main effect of treatment (*F* = 10.483, *p* = 0.001, *η*^*2*^: 0.512) with lower expression under LPS stimulation when compared with both other treatments.

For TLR-4 there was a main effect of treatment (*F* = 73.814, *p* < 0.001, *η*^*2*^: 0.881), significant differences between groups (*F* = 92.541, *p* < 0.001, *η*^*2*^*: *0.902), and statistically significant interactions (*F* = 24.478, *p* < 0.001, *η*^*2*^*:* 0.710). TLR-4 gene expression was higher in the High VO_2max_ group compared with the Low VO_2max_ group in all treatment conditions; in addition, within the same physical fitness group, under LPS stimulation, lower TLR-4 expression was observed when compared with both other treatments.

For AMPK, there was a main effect of treatment (*F* = 33.057, *p* < 0.001, *η*^*2*^: 0.768) and significant differences between groups (*F* = 5.930, *p* = 0.035, *η*^*2*^*: *0.372), with higher expression in the Low VO_2max_ group compared with the High VO_2max_ group, independently of treatment condition. For PGC-1α, there was a main effect of treatment (*F* = 45.989, *p* < 0.001, *η*^*2*^: 0.821), significant differences between groups (*F* = 10.532, *p* = 0.009, *η*^*2*^*: *0.513), and statistically significant interactions (*F* = 6.076, *p* = 0.009, *η*^*2*^*:* 0.378). PGC-1α was more highly expressed in the High VO_2max_ group compared with the Low VO_2max_ group in all treatment conditions; in addition, LPS + Rosiglitazone seemed to generate a higher expression when compared with the other treatments in the group with better physical fitness status. For PPAR-γ treated with other stimulus, there was a main effect of treatment (*F* = 27.859, *p* < 0.001, *η*^*2*^: 0.736), verifying higher expression under isolated Rosiglitazone and LPS + Rosiglitazone stimulations, when compared with LPS treatment, with isolated Rosiglitazone showing higher values when compared with LPS + Rosiglitazone.

## Discussion

Here, we showed for the first time that physical fitness status modulates the peripheral and cellular inflammatory response. We identified a positive relationship between LPS concentration and VO_2max_, independently of intensities of exercise sessions, as well as an association between LPS and cytokine concentrations, mainly IL-6 and IL-10 post-exercise. In addition, High VO_2max_ group exhibited higher PPAR-γ, TLR-4, IKK1, and PGC-1α gene expressions and lower IL-10 production in monocytes cell culture after LPS treatment. These results suggest that PPAR-γ pathway is highly expressed in trained individuals and this positive profile may act directly on the exercise-mediated peripheral response.

Middle or higher endotoxin concentrations at rest are related with unhealthy routine, such as sedentary lifestyle and poor nutrition, as well as with stress responses, such as after exercise training performed at higher volume, duration, and/or intensity. In the present study were evaluated LPS concentrations 1.5 h after breakfast, and found an augmented in endotoxin in higher VO_2max_ compared to low VO_2max_ individuals. These results may be explained, at least in part, by previous exercise-induced gastrointestinal symptoms ^30^ and total energy or nutrient intake ^31^, which may lead to higher LPS activity, suggesting that future studies should better control these parameters at rest. On the other hand, our findings are in agreement with previous studies conducted with exercised-animals (exercise training for 4 weeks with gradual increase in exercise training per week (12 m·min^−1^—21 m·min^−1^ for 60 min ) and athletes (triathletes) that observed smaller increases in LPS before an exercise session, when compared with untrained groups, suggesting a possible LPS tolerance in well-trained subjects^9,13^.

It is noteworthy to emphasize that LPS is a gut-derived bacterial endotoxin resulting from dysbiosis stimulated by hypercaloric/hyperlipidic diets, as well as sedentary behavior, favoring the activation of pro-inflammatory signaling pathways^32,33^. It is important to highlight that gut-microbiota act as an endocrine organ fully integrated in the host metabolism given its ability to produce and release biomarkers, as hormones and short chain fatty acids (SCFAs). Besides, its bacterial diversity change in response to several internal and external stimuli, as nutrition habits and physical exercise practice^34^.

Considering the gut-microbiota, it is well established in the literature that physical fitness status may directly influence the diversity/type and abundance of several intestinal bacteria and, in this context, it is suggested that regular physical exercise practitioners individuals have greater diversity and abundance of bacteria beneficial to health^35,36^. In this sense, according with gut-microbiome composition, the production and release of metabolites derived from microbial fermentation (i.e. butyrate, propionate and acetate) may be affected and these metabolites are able to regulate several cell and tissue functions given that these metabolites have pro and/or anti-inflammatory properties^37,38^.

In general, regular physical exercise practice acts as a protective behavior, modulating intestinal bacteria composition/diversity directly related to modulations in metabolism and immunity, especially to improve the SCFAs production and restore the intestinal epithelial barrier^39^. Indeed, other studies suggested that exercise training modulate Kupffer cells function enhancing the in vivo endotoxin clearance in animals, impacting directly the inflammatory response^40,41^. Thus, gut-microbiota and nutritional habits, directly associated with gut modulation, are determining factors for the regulation of the cell functionality and both parameters must be considered.

Changes in LPS concentration after an exercise session may directly, or indirectly, influence inflammatory cytokine release; our results showed that individuals with High VO_2max_ exhibited small changes in LPS concentrations post-exercise, however, we found higher IL-6 concentrations immediately after exercise performed at low and high intensities and lower IL-10 concentrations 60-min after low-intensity exercise in this group, suggesting that, inflammatory responses may be directly associated with physical fitness status, and that cytokine modulation in trained individuals occurs, at least in part, through LPS-independent mechanisms.

In this way, Ortega^42^ proposes that there are bioregulatory effects arising from exercise practice dependent on individual metabolic profile, given that in healthy individuals exercise training should stimulate an inflammatory response whereas in individuals with an installed inflammatory profile the same effort should mediate an anti-inflammatory response. According to some studies, physically active men and athletes have higher IL-6 mRNA expression in skeletal muscle after an exercise training session^43,44^, whereas higher IL-10 expression and release may be observed in individuals with an established inflammatory profile, such as sedentary and overweight individuals, as an attempt to inhibit an inflammatory condition represented by the high expression and release of inflammatory cytokines^45^.

It is important to highlight that IL-6 acts as an “energy sensor in response to decreased glycogen stores observed across effort sessions, increasing the bioavailability of energy substrate by glucose metabolism regulation via PI3-K/AKT, GLUT-4 activity and lipolysis^20,21^. On the other hand, as previously mentioned, IL-6 has a pleiotropic function, having anti and/or pro inflammatory properties. Considering its pro-inflammatory role, Peng and colleagues^46^ investigated the relationship between homocysteine, PPAR-γ and IL-6 gene expression in peripheral blood mononuclear cell cultures treated with PPAR-γ activators (troglitazone and rosiglitazone), and observed that homocysteine can promote the expression of inflammatory factors, such as IL-6. However, IL-6 concentrations were significantly reduced in PBMC treated with PPAR-γ activators culture supernatants, suggesting that the PPAR-γ activation can inhibit the pro-inflammatory factors production, mainly in population with inflammatory diseases. All these reports explain, at least in part, our findings regarding the cytokine concentrations in peripheral blood and monocyte culture supernatant, evidencing an intimate and dependent relationship between inflammatory responses, molecular activations and physical fitness status.

Our results seem to support the initial hypothesis that some molecular adaptations and mechanisms, mainly linked with PPAR-γ activation/signaling, may be imposed by long-term and regular exercise practice parallel with physical fitness status improvements which, at least in part, were proven when higher gene expression of TLR-4, IKK1, PGC-1α, and PPAR-γ was found in individuals with High VO_2max_, independently of monocyte phenotypic profile. Regarding TLR-4, differences were observed between physical fitness status and monocyte cell culture treatments, with higher expression in the High VO_2max_ group, especially under LPS + Rosiglitazone stimulation, suggesting an increase and/or immune response improvement against stressor stimuli which may be associated with adaptations imposed by exercise training. In this line, Nickel and colleagues^47^ showed that aerobic training (≤ 40 km/week for obese and ≥ 55 km/week for lean athletes) during 10-weeks induced an increase in the expression of TLR receptors, mainly TLR-4 and TLR-7, supporting our hypothesis that chronic exercise training may be able to modulate the TLR-activated axis.

Generally, TLR-4 activity is associated with inflammatory signaling, however its action is directly stress or agent and/or binder-dependent given that the TLR-4/MyD88 signaling pathway results in pro-inflammatory production and release, while the signal transduction by TLR-4/TRAM/TRIF/TRAF3 results in IFN-β production (related to innate immune response) and in anti-inflammatory cytokine release^48^. Therefore, TLR-4 may be associated with a better inflammatory response and, consequently, acts in a protective way on metabolism.

In this scenario, another important protein related to inflammatory response is IKK1 (or IKKα), a catalytic subunit of the IkB kinase complex that keeps NF-kB sequestered in the cytosol. In the present study we found higher expression of IKK1 in the High VO_2max_ group, when compared with the Low VO_2max_ group, leading us to hypothesize that a positive feedback occurs in the regulation of this protein in order to stimulate an alternative NF-kB pathway (maybe linked with IKK1-RelB activation).

Concerning PPAR-γ *upstream* marker signaling, PGC-1α is a co-activator of all PPAR isoforms, synergistically coordinating metabolic and inflammatory effects, such as AMPK-mediated mitochondrial regulation and expression of glucose membrane transport protein (GLUT-4)^49^, and insulin sensitivity^50^, as well as repressing inflammatory cytokines production through reduction in NF-kB phosphorylation^51^. According to Krämer and colleagues^52^, higher PGC-1α and PGC-1β expression as well as PPAR-α and PPAR-δ are observed in athletes (cyclists), when compared with physically active subjects; our findings corroborate these results given that High VO_2max_ group exhibited greater expression of the PPAR co-activator and was stimulus-independent, leading us to hypothesize that physical fitness status enables protective metabolic adaptations, mainly through imposing an anti-inflammatory environment even when conjugated pro and anti-inflammatory stimulus.

The positive modulation of the PGC-1α/PPAR-γ axis observed in the molecular environment may orchestrate the anti-inflammatory responses observed in the peripheral/systemic blood after acute aerobic exercise explains the circulating IL-6 and IL-10 concentrations. These findings allow us to understand that beneficial peripheral responses to exercise training are modulated directly by the molecular/cellular adaptations of "individual biological machinery" and a recent study conducted by Dorneles and colleagues^53^ corroborates and strengthens our hypothesis.

Considering the interesting findings of the present study, some limitations should be mentioned, such as the absence of information about relative and functional quantities of the main proteins investigated herein in order to verify if the gene expression is accompanied by a greater active and functional quantity of all proteins related to the PPAR-γ pathway. In addition, not having analyzed and explored other signaling pathways that could be in crosstalk with our experimental model, especially in cell culture with the drug combination, is an important limitation to be considered. Future studies should be conducted to answer these aforementioned gaps to better understand the metabolic mechanisms as well as the interactions between signaling pathways. Moreover, other studies should consider different populations, especially patients with an inflammatory profile, to identify the impacts of exercise training in the illness scenario as well as its possible ability to mediate reprogramming at the cellular level.

**Figure 7:**
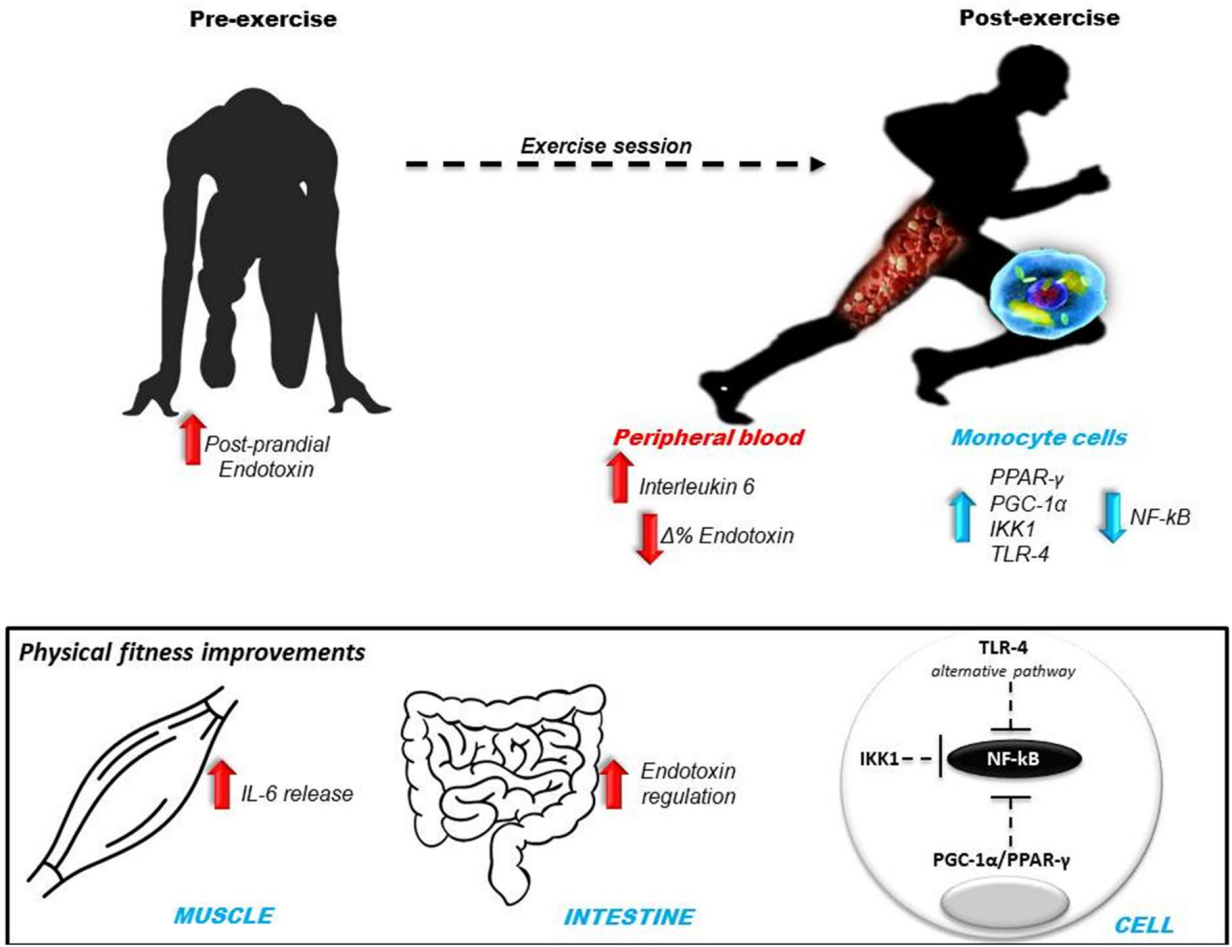
Conclusion diagram of the experiments and results.

In conclusion, even with elevated endotoxemia at rest 1.5 h after breakfast, individuals with greater VO_2max_ exhibited higher IL-6 concentration in peripheral blood post-acute aerobic exercise sessions, and lower IL-10 concentrations during recovery, evidencing that physical fitness status directly impacts the inflammatory responses through LPS-independent mechanisms. The anti-inflammatory effects associated with regular exercise training and, consequently, physical fitness status may be explained by the greater expression of proteins and cellular receptors, such as IKK1, TLR-4, and PGC-1α, in the group with High VO_2max_ displaying an extremely efficient cellular framework for fast and successful responses orchestrated by PPAR-γ (Fig. 7).
